# Implementation and Updating of Clinical Prediction Models: A Systematic Review

**DOI:** 10.1016/j.mcpdig.2025.100228

**Published:** 2025-05-23

**Authors:** Alexander Saelmans, Tom Seinen, Victor Pera, Aniek F. Markus, Egill Fridgeirsson, Luis H. John, Lieke Schiphof-Godart, Peter Rijnbeek, Jenna Reps, Ross Williams

**Affiliations:** a Department of Medical Informatics, Erasmus University Medical Center, Rotterdam, Netherlands; b Janssen Research and Development, Titusville, NJ

## Abstract

**Objective:**

To summarize the implementation approaches and updating methods of clinically implemented models and consecutively advise researchers on the implementation and updating.

**Patients and Methods:**

We included studies describing the implementation of prognostic binary prediction models in a clinical setting. We retrieved articles from Embase, Medline, and Web of Science from January 1, 2010, to January 1, 2024. We performed data extraction, based on Transparent Reporting of a Multivariable Prediction Model for Individual Prognosis or Diagnosis and Prediction Model Risk of Bias Assessment guidelines, and summarized.

**Results:**

The search yielded 1872 articles. Following screening, 37 articles, describing 56 prediction models, were eligible for inclusion. The overall risk of bias was high in 86% of publications. In model development and internal validation, 32% of the models was assessed for calibration. External validation was performed for 27% of the models. Most models were implemented into the hospital information system (63%), followed by a web application (32%) and a patient decision aid tool (5%). Moreover, 13% of models have been updated following implementation.

**Conclusion:**

Impact assessments generally showed successful model implementation and the ability to improve patient care, despite not fully adhering to prediction modeling best practice. Both impact assessment and updating could play a key role in identifying and lowering bias in models.

Clinical prediction models are evidence-based tools that can be used to personalize treatment through shared decision making, when implemented and communicated adequately.^1^ These models predict individualized risk of future outcomes based on multiple predictors and can be valuable tools for clinicians to identify high risk patients.^2^, ^3^, ^4^, ^5^ Consecutively, the clinician can recommend interventional treatment options and may prevent traumatizing outcomes. However, a low number of models is clinically implemented relative to the number of models developed.^6^ Therefore, we aimed to give recommendations on prediction modeling best practices.

Large observational health care databases, such as health care insurance claims or electronic health care records, often contain medical trajectories for thousands or millions of patients. These databases generally contain diverse patient populations, allowing the development of models for a large variety of prediction tasks.^7^ It is generally the consensus that the development of these models is performed as depicted in Figure 1, showing a pipeline for development to implementation of prediction models. An impact assessment is an assessment of the model effect in terms of patient care (including clinical outcomes). Despite many prediction models being developed, few make it to external validation, and even fewer get to implementation.^8^^,^^9^ This is problematic for futureproof shared decision making because the patient will expect a more personalized evidence-based approach in the future. Therefore, it is important to understand underlying contributing factors to limited model implementation. Previous work has considered different areas of improvement in model development: lack of clinical utility because many models are developed using poor task specification and design; complexity, when it comes to implementing a large model; and lack of widely used or trusted pipelines for model implementation.^10^, ^11^, ^12^, ^13^

**Figure 1:**
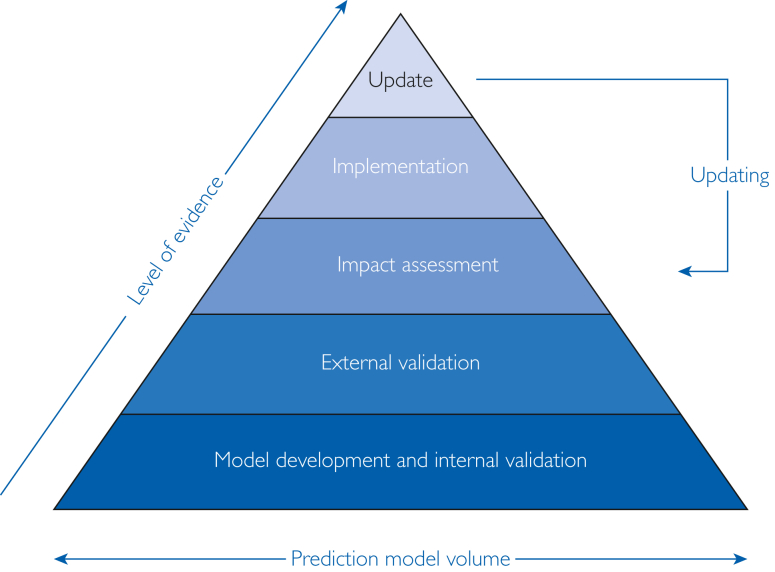
Pipeline of clinical prediction models. Model development and internal validation, assessment of performance in the same setting as the derivation data; external validation, assessment of performance in different database; impact assessment, assessment of performance in a clinical setting; implementation, eg, hospital information system, web-based application, and patient decision aid tool; update, simple (recalibration or revision), extension, meta-model, and dynamic.

There are various options for implementing clinical prediction models: integrating them into the hospital information system (HIS), using a web-based application or designing a patient decision aid tool. These options for implementing clinical prediction models sometimes overlap. Shifts in health care data and practices over time can lead to decreased model performance,^14^^,^^15^ necessitating regular updates.^4^^,^^5^^,^^7^

There are various types of updating strategies to maintain a model’s performance over time. First, simple updating consists of recalibration or revision, for example, deletion, and adjusts a model for novel data settings. Second, model extension incorporates new predictors to the model. Moreover, there are 2 relatively new updating modeling procedures. These are meta-modeling, combining multiple models into 1 meta-model and further updating it for a new data set, and dynamic, either periodic or discrete, updating.^13^ The optimal approach for updating remains unclear. There is a need for systematic reviews investigating implemented models that use some updating framework.

Previous systematic reviews on the implementation of clinical prediction models covered only 1 medical domain or disease, such as diabetes mellitus^9^ or cardiovascular disease risk prediction models.^8^ To our knowledge, no broad systematic review has been conducted on the implementation of clinical prediction models across diverse medical fields. We assume models are developed with the goal of implementing them at some point. Therefore, we view implementation of a model as a successful approach.

Although clinical prediction models have excited physicians for years, few prediction models have made it to implementation. A better understanding of the key barriers and facilitators of the implementation and updating of these models could improve their transportability into a health care setting. Therefore, this systematic review aimed to summarize successful approaches and updating methods used in clinical settings and advise researchers on best practices.

## Patients and Methods

This systematic review was registered with PROSPERO (international prospective register of systematic reviews), code CRD42024506783,^16^ and adheres to the Preferred Reporting Items for Systematic Reviews and Meta-Analyses (PRISMA)^17^ (Supplemental Appendix 1, available online at https://www.mcpdigitalhealth.org/) and Transparent Reporting of a Multivariable Prediction Model for Individual Prognosis or Diagnosis-Systematic Review and Meta-Analyses (TRIPOD-SRMA)^18^ (Supplemental Appendix 2, available online at https://www.mcpdigitalhealth.org/) guidelines. No separate protocol exists beside the PROSPERO protocol. Compared with what was registered in PROSPERO, no changes were made to the methodology. There was no funding source for this individual study.

### Search Strategy

An information specialist was consulted to formulate the search queries in line with the PRISMA-S guideline^19^ and included the terms listed in Supplemental Appendix 3 (available online at https://www.mcpdigitalhealth.org/). We performed a search strategy consisting of 3 clauses that incrementally narrowed down the search results: *Prediction AND Models AND Implementation*. The articles were retrieved from the Embase, Medline, and Web of Science databases on January 26, 2024. The search was limited to articles published between January 1, 2010, and January 1, 2024, written in English, Spanish, French, or German. A cutoff of 2010 was chosen to only consider the more recent modeling practices. Studies that were not original research (eg, editorials), and animal studies were excluded.

### Selection Criteria

The eligibility criteria for title, abstract, and full-text screening were as follows:

I. Clinically implemented prognostic prediction models.
  • All medical domains and all methods of implementation into clinical use, for example, integration in HIS, web-based application, mobile application, usage in multidisciplinary meeting, integration in a guideline, and MD Calc.
  • Limited to prognostic prediction (predicting a future outcome for a patient), no diagnostic prediction (predicting a diagnosis of an already present disease). External validation alone was not sufficient for inclusion.
II. The use of a binary classifier that predicts whether an outcome occurs within a defined time-at-risk, for example, generalized linear models, logistic regression, random forest, gradient boosting machine, K-nearest neighbors, naive Bayes, AdaBoost, decision tree, multilayer perceptron, and deep learning. Survival models that predict when an outcome occurs were excluded.

### Screening and Data Extraction

Articles were screened by 2 reviewers independently: 1 author (A.S.) and 1 of the other coauthors (T.S., V.P., A.M., E.F., H.J., J.R., R.W.) screened 1 of the 7 equal parts. Title and abstract screening was managed with ASReview,^20^ an online artificial intelligence–based systematic review platform. All potentially relevant records were screened. In case of disagreement, we included the article for the full-text screening. Consecutively, full-text screening took place. The data extraction was performed using a standardized data capture Excel file by the main researcher (A.S.) in line with the TRIPOD statement,^21^ the Checklist for critical appraisal and data extraction for systematic Reviews of prediction Modeling Studies (CHARMS),^22^ and the Prediction model Risk of Bias Assessment Tool (PROBAST).^23^ TRIPOD is a guideline on the reporting of prediction models. Applicability, whether population, predictors, or outcomes of the study differ from those specified in the review question, within PROBAST is omitted because this systematic review was performed across all medical domains. The data capture file contained items from these 3 checklists together with other items we deemed relevant. A full list of items extracted, including the guideline/checklist the items were extracted from or other reason, is included in Supplemental Appendix 4 (available online at https://www.mcpdigitalhealth.org/). Data were captured from the main text, abstract, and supplemental material if available. Information from previous studies to the included implementation studies, describing model development, internal validation, and external validation, was also included when insufficient information was provided in the publication describing their implementation. We extracted data on several categories for each model in each study as follows:

• General characteristics: machine learning technique, use of multidisciplinary teams, intended user of the model, setting, clinical specialty, continent of origin, and impact factor
• Adherence to TRIPOD reporting guideline characteristics: demographic characteristics of the development data, number of features, external validation (study design), area under the curve (AUC), and calibration assessment
• PROBAST risk of bias assessment: data source, events per predictor (EpP), and handling of missing data
• Implementation and updating strategies: means of implementation, impact assessment (study design), improvement of care in impact assessment, and updating (strategy)

Clinical improvement in impact assessment was defined as a statistically significant effect.

### Data Synthesis

Owing to heterogeneity of prediction models, we were unable to perform a meta-analysis. Descriptive analyses were performed: frequencies and percentages for categorical variables and mean and SD or median and IQR for continuous variables. Results were visualized when applicable. All statistical analyses were performed in R 4.1.1 (R Foundation for Statistical Computing). The R code has been published on GitHub.

## Results

### Literature Search

Of the 1872 unique publications identified in the literature search (Supplemental Appendix 5, available online at https://www.mcpdigitalhealth.org/), 108 full-text publications were assessed for eligibility following title and abstract screening. A total of 37 publications met the eligibility criteria after full-text screening.^24^, ^25^, ^26^, ^27^, ^28^, ^29^, ^30^, ^31^, ^32^, ^33^, ^34^, ^35^, ^36^, ^37^, ^38^, ^39^, ^40^, ^41^, ^42^, ^43^, ^44^, ^45^, ^46^, ^47^, ^48^, ^49^, ^50^, ^51^, ^52^, ^53^, ^54^, ^55^, ^56^, ^57^, ^58^, ^59^, ^60^ The included publications contained 56 prediction models (5 publications described the implementation of 2 models and 7 articles described the implementation of 3 models). Table 1 depicts the main characteristics of the included publications and consecutive models. A list of studies excluded at full-text screening stage, including brief reasons, is provided in Supplemental Appendix 6 (available online at https://www.mcpdigitalhealth.org/).

**Table 1:** Details of the Included Clinical Prognostic Binary Prediction Models

Reference, year	Country	Clinical specialty	MDT (yes/no)	Demographic model development and internal validation	Modeling method	Outcome	Outcome (%)	AUC (95% CI)	Calibration (yes/no, plot)	AUPRC and/or DCA	External validation (yes/no, prospective)	Impact assessment (yes/no, design)	Implementation strategy	Updating (yes/no, method)
Agius et al,^24^ 2023	Denmark	Hematology	Yes	N=3720, 39.8% women	Other ensemble	Severe infection and/or CLL treatment	NA	0.78 (0.69-0.86)	No	Neither	Yes	Yes, ppi	HIS	No
Chang et al,^25^ 2024	Taiwan^a^	Emergency medicine	Yes	N=8274, mean age 56.1 (SD 16.7) y, 32.3% women, BMI 23.8 (SD 4.5)	LightGBM	Sepsis	4.0	0.961	No	Neither	No	Yes, retrospective	HIS	No
				N=8274, mean age 56.1 (SD 16.7) y, 32.3% women, BMI 23.8 (SD 4.5)	LightGBM	ICU admission	4.4	0.973	No	Neither	No	Yes, retrospective	HIS	No
				N=8274, mean age 56.1 (SD 16.7) y, 32.3% women, BMI 23.8 (SD 4.5)	XGBoost	Death	2.5	0.975	No	Neither	No	Yes, retrospective	HIS	No
Choi et al,^26^ 2023	South Korea	Emergency medicine	No	N=23,550, mean age 62.7 y, 45.3% women	Bayesian neural network	Bacteremia result	10.7	0.754 (0.737-0.771)	Yes, plot	Neither	Yes	Yes, ppi	HIS	No
Cronin et al,^27^ 2014	USA	General	No	N=45,924	Logistic regression	Hospital readmission	12.1	0.705 (0.697-0.713)	Yes, plot	Neither	No	Yes, prospective	HIS	No
Dontchos et al,^28^ 2022	USA	Radiology	No	N=39,272, mean age 57.1 y, 100% women	Deep convolutional neural network	Mammographic breast density	NA	NA	No	Neither	No	Yes, ppi	HIS	No
Esbenshade et al,^29^ 2020	USA	Oncology	No	N=463, 36.0% women	Logistic regression	Bloodstream infection	19.7	0.898	No	Neither	Yes	Yes, prospective	Web application	Yes, deletion
				N=463, 36.0% women	Logistic regression	Bloodstream infection	19.7	0.895	No	Neither	Yes	Yes, prospective	Web application	Yes, deletion
Fenn et al,^30^ 2021	USA	Emergency medicine	No	N=468,167	LightGBM	Inpatient unit admission	13.5	0.873 (0.871-0.874)	No	AUPRC 0.636 (0.631-0.640)	Yes	Yes, prospective	HIS	No
				N=468,167	LightGBM	ICU admission	1.7	0.951 (0.948-0.954)	No	AUPRC 0.461 (0.443-0.478)	Yes	Yes, prospective	HIS	No
Giannini et al,^31^ 2019	USA	Emergency medicine	No	N=171,710, mean age 58.0 y, 50.4% women, BMI 28.7	Random forest	Severe sepsis and septic shock	NA	0.88 (0.85-0.91)	No	Neither	No	Yes, ppi	HIS	No
Grout et al,^32^ 2021	USA	Cardiology	No	N=98,324, mean age 66.5 y, 55.0% women	Logistic regression	Undiagnosed atrial fibrillation	0.589	0.806 (0.802-0.810)	No	Neither	No	Yes, pilot	HIS	No
de Holanda et al,^33^ 2023	Brazil	Internal medicine	No	N=879,832	GBM	Death	0.163	0.89	No	Neither	Yes	No	Web application	No
				N=879,832	GBM	Hospital admission	0.0826	0.75	No	Neither	Yes	No	Web application	No
Hsu et al,^34^ 2023	Taiwan^a^	Emergency medicine	Yes	N=2666, mean age 65.3 (SD 16.9) y, 45.7% women, BMI 23.0 (SD 4.8)	Multilayer perceptron	Sepsis	31.7	0.852	Yes, plot	Neither	No	Yes, retrospective	HIS	No
				N=2666, mean age 65.3 (SD 16.9) y, 45.7% women, BMI 23.0 (SD 4.8)	Multilayer perceptron	ICU admission	6.0	0.743	Yes, plot	Neither	No	Yes, retrospective	HIS	No
				N=2666, mean age 65.3 (SD 16.9) y, 45.7% women, BMI 23.0 (SD 4.8)	Multilayer perceptron	Death	12.8	0.796	Yes, plot	Neither	No	Yes, retrospective	HIS	No
Hulsbergen et al,^35^ 2022	Netherlands	Neurosurgery	No	N=1062, 57.9% women	Logistic regression	Death	26.1	0.709	Yes, plot	Neither	No	No	Web application	No
Jauk et al,^36^ 2020	Austria^a^	Internal medicine	Yes	N=8561	Random forest	Delirium	NA	0.9095	No	Neither	No	Yes	HIS	No
				N=19,000	Random forest	Delirium with a history of delirium	NA	0.854 (0.843-0.864)	Yes, plot	Neither	No	Yes, prospective	HIS	No
				N=19,000	Random forest	Alcohol withdrawal delirium	NA	0.929 (0.912-0.946)	Yes, plot	Neither	No	Yes, prospective	HIS	No
Jauk et al,^37^ 2019	Austria^a^	Intensive Care	Yes	N=61,864, mean age 56.8 y, 51.1% women	Random forest	ICU admission	7.6	0.911 (0.903-0.919)	No	Neither	Yes	Yes, prospective	HIS	No
Jeon et al,^38^ 2023	South Korea	Nephrology	No	N=823, mean age 45.2 y, 51.6% women	Other ensemble (RF and LR)	Fair renal adaptation	86.7	0.846 (0.762-0.930)	No	AUPRC 0.965 (0.944-0.978)	No	No	Web application	No
				N=823, mean age 45.2 y, 51.6% women	Other ensemble (RF and LR)	Good renal adaptation	61.3	0.626 (0.541-0.712)	No	AUPRC 0.709 (0.647-0.788)	No	No	Web application	No
Karabacak and Margetis,^39^ 2023	USA	Neurosurgery	No	N=3073, 51.4% women	Random forest	Prolonged length of stay	24.5	0.760	Yes, plot	AUPRC 0.586	No	No	Web application	No
				N=3073, 51.4% women	Random forest	Nonhome discharge	23.4	0.719	Yes, plot	AUPRC 0.402	No	No	Web application	No
				N=3073, 51.4% women	Random forest	Major complication	12.3	0.749	Yes, plot	AUPRC 0.318	No	No	Web application	No
Kilpatrick et al,^40^ 2014	USA	Internal medicine	No	N=3028	Logistic regression	Severe hypoglycemia	5.7	0.697	No	Neither	No	Yes, prospective	HIS	No
Kiss et al,^41^ 2022	Hungary	Internal medicine	No	N=2387, 43.2% women	XGBoost	Acute necrotizing pancreatitis	9.76	0.757	No	Neither	No	No	Web application	No
Koppes et al,^42^ 2021	Netherlands	Obstetrics and Gynecology	No	N= 515, mean age 32.2 (SD 5.0) y, 100% women, BMI 25.3 (SD 5.7)	Logistic regression	Succesful vaginal birth after cesarean	72.0	0.708 (0.686-0.729)	Yes, plot	Neither	No	Yes, ppi	Patient decision aid	No
Kumar et al,^43^ 2022	Singapore	Obstetrics and Gynecology	No	N=222, mean age 30.5 (SD 3.1) y, 100% women, BMI 23.2 (SD 4.5)	Other ensemble (GBM and SVM)	Gestational diabetes mellitus	13.1	0.93	No	Neither	No	No	Web application	No
Lazebnik et al,^44^ 2022	United Kingdom	Urology	No	N=723, mean age 61.2 y, 62.5% women	Random forest	Acute kidney injury	32.0	0.75	No	Neither	No	No	Web application	No
Levin et al,^45^ 2020	USA	General	No	N=15,613	Random forest	Hospital discharge	NA	NA	No	Neither	No	Yes, ppi	HIS	Yes
Li et al,^46^ 2022	Taiwan^a^	Anesthesiology	Yes	N=4448, mean age 65.3 y, 57.6% women	Logistic regression	Adverse event	2.3	0.794 (0.718-0.869)	No	Neither	No	Yes, ppi	HIS	No
				N=4448, mean age 65.3y, 57.6% women	Logistic regression	ICU admission	3.6	0.856 (0.804-0.908)	No	Neither	No	Yes, ppi	HIS	No
				N=4448, mean age 65.3 y, 57.6% women	Random forest	Prolonged length of stay	9.0	0.854 (0.818-0.890)	No	Neither	No	Yes, ppi	HIS	No
Liu et al,^48^ 2018	USA	Oncology	Yes	N=2824, 0% women	Absolute risk	Colorectal cancer	45.6	NA	No	Neither	Yes, prospective	No	Patient decision aid	No
				N=2272, 100% women	Absolute risk	Colorectal cancer	45.6	NA	No	Neither	Yes, prospective	No	Patient decision aid	No
Liu et al,^47^ 2023	Taiwan^a^	Nutrition	Yes	N=8411, mean age 59.9 (SD 12.2) y, 46.8% women, BMI 26.0 (SD 4.3)	XGBoost	HbA1c < 7%	37.7	0.738	No	Neither	No	No	HIS	No
Lupei et al,^49^ 2022	USA	Emergency medicine	Yes	N=2041, 51.1% women	Logistic regression	Severe COVID-19	24.3	0.87 (0.83-0.91)	No	Neither	Yes	Yes, prospective	HIS	No
Major and Aphinyanaphongs,^50^ 2020	USA	Palliative care	No	N=94,733, 60.1% women	Random forest	Death	0.042	0.872 (0.861-0.882)	Yes, plot	Neither	No	Yes, prospective	HIS	No
Saglietto et al,^51^ 2023	EU	Cardiology	No	N=3128, mean age 58.1 (SD 10.3) y, 31.3% women, BMI 28.4 (SD 4.5)	Random forest	Arrythmic recurrence after catheter ablation	25.8	0.721 (0.680-0.764)	Yes, plot	Neither	No	No	Web application	Yes, Platt scaling recalibration
Schrempf et al,^52^ 2022	Austria^a^	Cardiology	Yes	N=128,000	Random forest	Major adverse cardiovascular events	22.9	0.879	Yes, plot	Neither	No	No	HIS	No
Shah et al,^53^ 2020	USA	General	No	N=157,958, mean age 70.6 y, 54.8% women	Logistic regression	Death	11.8	0.913 (0.908-0.918)	No	Neither	No	Yes, prospective	HIS	Yes, recalibration
				N=152,192, 54.8% women	Logistic regression	Death	1.9	0.710 (0.706-0.715)	No	Neither	No	Yes, prospective	HIS	Yes, recalibration
				N=147,731, 54.8% women	Logistic regression	All-cause readmission	11.5	0.85 (0.84-0.87)	No	Neither	No	No	HIS	Yes, recalibration
Solomon et al,^54^ 2023	USA	Rheumatology	No	N=16,336, mean age 55.1 y, 77.7% women	Logistic regression	Telehealth appropriateness	15.7	0.843 (0.819-0.866)	No	Neither	Yes	Yes, pilot	Web application	No
Starr et al,^55^ 2021	USA	General surgery	No	N=2451	Other ensemble	Death	2.17	0.94	No	AUPRC 0.377	No	Yes, retrospective	HIS	No
Syed and Khan,^56^ 2020	Saudi Arabia	Internal medicine	No	N=4896	Decision forest	Type II diabetes mellitus	20.2	0.867	Yes	Neither	Yes	No	Web application	No
Tammemägi et al,^57^ 2021	Canada	Oncology	Yes	N=14,576, 38.6% women, BMI 28	Logistic regression	Lung cancer	1.39	0.772 (0.743-0.799)	Yes, plot	Neither	Yes	Yes, pilot	Web application	No
Wang et al,^58^ 2022	South Korea	Internal medicine	No	N=19,159, mean age 62.3 (SD 11.7) y, 44.3% women, BMI 25.2 (SD 4.2)	XGBoost	End-stage renal disease	8.3	0.947 (0.944-0.950)	Yes, plot	DCA, AUPRC 0.785	No	No	HIS	No
Wang et al,^59^ 2023	China	Neurology	Yes	N=645, 40.8% women	Random forest	Stroke recurrence	13.0	0.946	No	Neither	No	No	Web application	No
Yeh et al,^60^ 2023	Taiwan^a^	Plastic surgery	Yes	N=224, mean age 45.8 (SD 20.3) y, 33.9% women, BMI 24.5 (SD 4.8)	Random forest	Graft surgery	44.6	0.757	No	Neither	No	Yes, retrospective	HIS	No
				N=224, mean age 45.8 (SD 20.3) y, 33.9% women, BMI 24.5 (SD 4.8)	XGBoost	Prolonged hospital stay	57.6	0.815	No	Neither	No	Yes, retrospective	HIS	No
				N=224, mean age 45.8 (SD 20.3) y, 33.9% women, BMI 24.5 (SD 4.8)	LightGBM	Overall adverse effects	61.6	0.845	No	Neither	No	Yes, retrospective	HIS	No

AUC, area under the curve; AUPRC, area under the precision-recall curve; BMI, body mass index (in kg/m^2^); DCA, decision curve analysis; EU, European Union; GBM, gradient boosting machine; HIS, hospital information system; LightGBM, light gradient boosting machine; LR, logistic regression; MDT, multidisciplinary team; ppi, preintervention and postintervention study; RF, random forest; SVM, support vector machine; XGBoost, eXtreme Gradient Boosting.

a Prediction models developed by 2 hospital groups.

### General Characteristics

**Figure 2:**
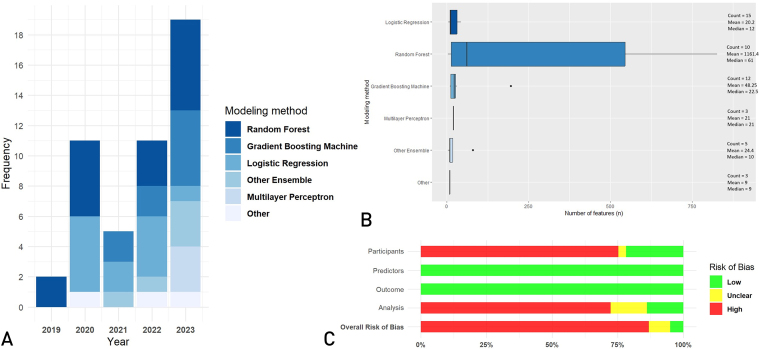
(A) Trend in modeling methods, publication year of implementation article. (B) Boxplots of number of features per modeling method. (C) PROBAST risk of bias assessment of the included prediction model studies. Other—2 absolute risk models, 1 Bayesian neural network, 1 deep convolutional neural network, and 1 decision forest model. One random forest number of feature outlier of 9614 features is not depicted in (B).

The most frequently used machine learning techniques are random forest (16 [29%]), followed by logistic regression (15 [27%]), gradient boosting machine (12 [21%]), other ensemble (5 [9%]), and multilayer perceptron (3 [5%]). Figure 2A depicts the trend in modeling methods. Multidisciplinary teams often (24 [43%]) worked together to develop the models. Intended users of the models were predominantly physicians (22 [39%]), followed by health care professionals (13 [23%]), patients (3 [5%]), and nurses (1 [2%]). Seventeen [30%] models did not mention an intended user. Included models were developed in a primary (5 [9%]), secondary (23 [41%]), secondary/tertiary (12 [21%]), or tertiary (16 [29%]) setting. The most common clinical specialties these models were focused on are emergency medicine (11 [20%]), internal medicine (9 [16%]), oncology (5 [9%]), neurosurgery (4 [7%]) and cardiology (3 [5%]). Researchers from North America described the implementation of models most frequently (23 [41%]), followed by Asia (20 [36%]), Europe (11 [20%]), and South America (2 [4%]). The publications were published in journals with a median impact factor of 3.8 (IQR, 2.0).

### Adherence to TRIPOD Reporting Guideline Characteristics

The demographic characteristics of the data used to develop and validate the included models are shown in Supplemental Appendix 7 (available online at https://www.mcpdigitalhealth.org/). Boxplots of the number of features per model are shown by Figure 2B. External validation was performed in 15 (27%) models. It was performed prospectively in 2 [4%] models. Seven [13%] models provided the programming code to the reader. The mean AUC for model development and internal validation was 0.814 (SD, 0.135). For external validation, the mean AUC was 0.772 (SD, 0.092). When it comes to impact assessment studies, the mean AUC was 0.821 (SD, 0.067). Calibration was evaluated in 18 (32%) of the model development and internal validation studies, with 17 (94%) using calibration plots. For external validation, 6 (40%) evaluated calibration, all using calibration plots. In impact assessments, 11 (29%) included calibration assessments, with 8 (73%) using calibration plots.

### PROBAST Risk of Bias Assessment

The overall risk of bias was high in 32 (86%) publications. The PROBAST risk of bias assessment sources of the included studies are presented in Figure 2C and Supplemental Appendix 8 (available online at https://www.mcpdigitalhealth.org/). The most important contributing domains to this high risk of bias were the participant and analysis domains. The high risk of bias in the participant domain was predominantly caused by the use of observational health data. When it comes to data sources used in the participants domain of PROBAST, observational health data were mostly used (40 [71%]), followed by retrospective registries (9 [16%]), retrospective and prospective registries (4 [7%]), and prospective registries (2 [4%]), and, finally, 1 [2%] nested case–control study. The most important contributing factors to the high risk of bias in the analysis domain were insufficient performance metrics, low EpP, and not handling missing data adequately. For the analysis domain of PROBAST, the EpP were summarized into 3 categories, ranging from zero to 10 (14 [25%]), 10 to 20 (5 [9%]), and greater than 20 EpP (28 [50%]). For 9 [16%] models, it was not possible to calculate the EpP. Moreover, 19 [34%] models performed imputation.

### Implementation and Updating Strategies

**Figure 3:**
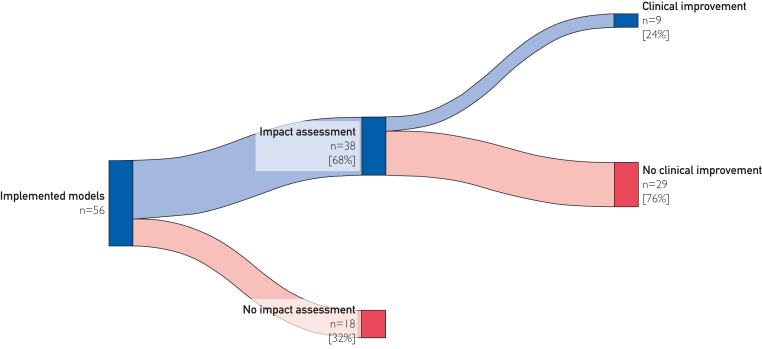
Sankey diagram of implementation, impact assessment, and clinical improvement of models.

In total, 37 (100%) publications dealt with model implementation and 4 (11%) publications with model updating. When it comes to the means of implementation, 35 (63%) models were integrated into the HIS, 18 (32%) models were implemented in a web-based application, and 3 (5%) models were integrated into a patient decision aid tool. Impact assessment in any form was performed in 38 (68%) models. Figure 3 depicts a Sankey diagram of how many of the included models performed an impact assessment and whether this assessment showed clinical improvement. For the impact assessment of the models, different study designs were applied: 15 (39%) were prospective impact assessments, 11 (29%) were retrospective impact assessments, 8 (21%) were preintervention and postintervention studies, 4 (11%) were pilot assessments, and 4 (11%) looked into the satisfaction of users. Eighteen (32%) models described only implementation and did not perform a study on the impact of implementation. Supplemental Appendix 9 (available online at https://www.mcpdigitalhealth.org/) describes which and how impact assessments showed an improvement in care. Seven (13%) models were updated. One model reported on the time from model development to updating (2 years). The other updated models did not report on this. Of the updated models, the updating strategies consisted of 4 (57%) recalibrations and 2 (29%) deletions. One (14%) model did not report on the updating strategy.

## Discussion

Prediction models may improve patient treatment but few developed models get implemented.^8^^,^^9^ We aimed to summarize successful approaches and updating methods used in clinical settings for implemented clinical prediction models and subsequently advise researchers on best practices. Our results suggest that implemented models often have a high risk of bias based on PROBAST and may indicate lack of adherence to TRIPOD reporting and lack of a model updating strategy. Despite not fully adhering to prediction modeling best practices, the impact assessments consistently highlighted the successful implementation of the models and may therefore demonstrate their potential to enhance patient care. On the basis of this, a table with recommendations has been depicted in Table 2.

**Table 2:** Recommendations for the Prediction Model Pipeline

Recommendations	Additional recommendations
Following modeling best practice	• Determine whether there is a clinical need • Report in line with TRIPOD • Include AUROC, AUPRC and calibration plot, and decision curve analysis • Prospectively register a model
Impact assessment plan	• Well-designed impact study • Include AUROC, AUPRC, calibration plot, and decision curve analysis
Implementation plan	• Establish multidisciplinary team with focus on clinical prediction models • Efficient integration into workflow • Publish with standardized terminology
Model updating plan	• When to update • How to update

Registering a model prospectively is currently not possible.

AUROC, area under the receiver operating characteristic curve; AUPRC, area under the precision-recall curve.

### Bias in Observational Health Data

Most implemented models are associated with a high overall risk of bias according to PROBAST. The use of observational health data for model development and external validation, within the participant domain, is the main culprit for 86% of models being high risk of bias in this study.^23^ This finding is partially reinforced by Andaur Navarro et al.^61^ However, an argument for using observational health data is that this is often the domain in which these models are applied. Developing and evaluating the model in this setting therefore seems more appropriate. Prospective data collection, a means of collecting data that is not deemed at high risk of bias in contrast to observational health data, in a highly controlled environment could create bias because it does not represent the real-world scenario. Moreover, there are many situations in which prospective data collection is not an option. For these reasons, we believe more research on large observational databases is needed to identify the actual risk of bias in different settings, instead of labeling these type of data as high risk by default. We believe that a proper impact assessment is needed for all models to assess the level of bias. This is not dependent on the source of the data. However, we found no pragmatic randomized controlled trials (RCTs) analyzing patient care processes and/or outcomes. Model updating should be considered for models with high risk of bias. Our review suggests that updating strategies, however, are infrequently (13%) included in the modeling pipeline. We recommend that researchers always provide an updating strategy that addresses how often they will update and how they will update (updating method and data).

### Bias Owing to Calibration, Sample Size, and External Validation

Other characteristics responsible for a high risk of bias are mentioned in both the analysis domain of PROBAST and the TRIPOD statement. First, our results suggest that the implemented models assessed calibration in only one-third of models. This is comparable with the relative number of calibration assessments in other systematic reviews.^62^, ^63^, ^64^, ^65^ The literature has shown that assessing calibration is essential in validating models. Consider a model X (AUC, 0.78; well calibrated) and model Y (AUC, 0.9; which adds 10% risk to everyone with a low risk). A patient with a true risk of 0.1% would be predicted a risk approximately 0.1% by model X, but 10.1% by model Y. If the threshold for intervention is 10+%, then the patient with a true low risk of 0.1% would incorrectly be identified as high risk by model Y. Second, a low number of EpP resulted in high risk of bias. This is partly because of an unclear cutoff point. A categorization is made of 0-10, 10-20, and more than 20, with 10-20 EpP being a gray area. Apart from there being a gray area, this way of calculating a sample size would be adequate for regression models. However, for other machine learning algorithms, different sample size calculations might be a better fit. Whittle et al^66^ describes an alternative way of calculating sample size for machine learning models. Another means to control bias is by performing external validation. However, our review may indicate there is a lack (27%) of external validation before implementation. This is also in line with the results of other systematic reviews.^62^^,^^64^^,^^65^ External validation is vital because it provides insights into the impact of missing predictors and the accuracy of the model across different patient characteristics.^67^ A classic example that illustrates how important external validation is before implementation of a model is the EPIC machine learning model. This model was found to perform poorly in external validation following its implementation across many US hospitals.^68^

### Model Implementation

When investigating what may increase the chance of successful model implementation, we found that implementation of models may be helped by the use of parsimonious models and having a strategy to directly implement into the local HIS. Most of the models conformed to some sort of parsimony. Parsimonious models have the advantages of being easier to grasp by clinicians and to lower the difficulty of integration into the local HIS. When it comes to having a plan to directly implement into the local HIS, what stood out is that 2 hospital groups have been quite successful at implementing models, comprising 25% of all implemented models.^25^^,^^34^^,^^36^^,^^37^^,^^47^^,^^52^^,^^60^ Notably, these organizations have established multidisciplinary teams with a focus on clinical prediction models early on consisting of medical domain experts, data scientists, and HIS experts, which hold meetings frequently to discuss progress in the prediction model pipeline.

### Strengths and Limitations

This systematic review has several strengths. Our extensive literature search over a prolonged period yielded numerous eligible studies across various clinical settings. Moreover, the multidisciplinary team of researchers, with backgrounds in medicine, informatics, pharmacy, and econometrics, allows for a plurality of perspectives.

However, there are also several limitations. First, there could be publication bias where implemented models that fail to make an impact are not published and therefore are not captured in this review. Second, lack of standardized terminology (eg, not using the term implementation) in the title or abstract of publications describing implementation, prevented us from finding them. Not finding these articles through our search might have resulted in selection bias. Third limiting factor is the heterogeneity of studies. This heterogeneity prevented the possibility to perform a meta-analysis. In addition, not having a second reviewer verifying the extracted data and PROBAST assessments might have caused single-reviewer bias. Because the TRIPOD-AI statement was not available at the time of data extraction, it was omitted. Finally, in this study, we considered model implementation as a success owing to few models being implemented. We wanted to see what the barriers are to model implementation because that is the first step toward impact. However, in future research, as the barriers to implementation improve, it would be useful to investigate strategies that lead to positive impact to medical care by an implemented model. In this article, clinical improvement was defined as a statistically significant effect. However, many impact assessments may not have been powered enough to reach significance. This may indicate better guidelines for impact assessment need to be developed.

### Future Work

There are thorough guidelines, TRIPOD and PROBAST, on prediction modeling best practices and reporting. However, researchers seem to not entirely comply with them. We suspect this to be caused by a barrier that researchers face in the real-world. Researchers do not always have access to RCT or prospective data. However, there is still a need for clinical prediction models in these areas of medicine. Critical appraisal and reporting standards might be too stringent and should accommodate this real-world barrier more in the future. When combating the assignment of a high risk of bias to a model, performing impact assessments and updating models is essential. Further, best practice when it comes to updating has sparsely been described in literature. Therefore, new insights into updating best practices would be essential to make models futureproof.

### Recommendations

When researchers within an institution wish to implement a model, we recommend the creation of an implementation plan early on. This is likely to be aided by an impact assessment and an updating strategy. In addition, we think there should be future research to assess the risk of bias for models developed and validated on observational health data. Our recommendations are as follows: (1) to follow prediction modeling best practices by ensuring the prediction model can answer a clinically meaningful question, ensuring reporting transparency and following guidelines for model development/validation ensuring calibration is assessed; (2) to make implementation feasible by having a multidisciplinary team that includes medical domain experts, data scientists, and HIS experts who know how to implement it efficiently into an HIS system and publish with standardized terminology; (3) to have a well-designed impact assessment, preferably a pragmatic RCT impact assessment^42^; and (4) to formulate a model updating strategy that specifies when and how to update the model.

## Conclusion

Successfully implemented models have often performed impact assessments, demonstrating that models can improve patient care, despite model development and validation not fully adhering to prediction modeling best practices. Because implemented models rarely had updating strategies and literature on it is sparsely available, updating should be performed more frequently and future research should focus on best practice guidelines for model updating.

## Potential Competing Interests

Dr Reps is an employee and shareholder of Johnson & Johnson. All authors work for a research group at Erasmus University Medical Center that receives/received an unconditional grant for methodological research by Johnson & Johnson. The grant is for the institute.

## Ethics Statement

This study, a systematic review, is exempt from an ethics statement as it is not a study analyzing patient-level data. The individual studies included had to request their own ethics approval.

## References

[1] Stiggelbout A.M., van der Weijden T., de Wit M.P. (2012). Shared decision making: really putting patients at the centre of healthcare. BMJ.

[2] Harrell F.J. (2001).

[3] Moons K.G.M., Royston P., Vergouwe Y., Grobbee D.E., Altman D.G. (2009). Prognosis and prognostic research: what, why and how?. BMJ.

[4] Steyerberg E. (2009).

[5] Steyerberg E.W., Moons K.G., van der Windt D.A. (2013). Prognosis Research Strategy (PROGRESS) 3: prognostic model research. PLoS Med.

[6] van de Sande D., van Genderen M.E., Huiskens J., Gommers D., van Bommel J. (2021). Moving from bytes to bedside: a systematic review on the use of artificial intelligence in the intensive care unit. Intensive Care Med.

[7] Vaid A., Sawant A., Suarez-Farinas M. (2023). Implications of the use of artificial intelligence predictive models in health care settings: a simulation study. Ann Intern Med.

[8] Damen J.A., Hooft L., Schuit E. (2016). Prediction models for cardiovascular disease risk in the general population: systematic review. BMJ.

[9] Collins G., Mallett S., Omar O., Yu L.M. (2011). Developing risk prediction models for type 2 diabetes: a systematic review of methodology and reporting. BMC Med.

[10] Moorthie S. (2021). What is clinical utility? PHG Foundation. https://www.phgfoundation.org/explainer/clinical-utility.

[11] Vickers A.J., van Calster B., Steyerberg E.W. (2019). A simple, step-by-step guide to interpreting decision curve analysis. Diagn Progn Res.

[12] Steyerberg E.W., Vickers A.J., Cook N.R. (2010). Assessing the performance of prediction models: a framework for traditional and novel measures. Epidemiology.

[13] Binuya M.A.E., Engelhardt E.G., Schats W., Schmidt M.K., Steyerberg E.W. (2022). Methodological guidance for the evaluation and updating of clinical prediction models: a systematic review. BMC Med Res Methodol.

[14] Castillo D. (2021). What is covariate shift?. Seldon.

[15] Finlayson S.G., Subbaswamy A., Singh K. (2021). The clinician and dataset shift in artificial intelligence. N Engl J Med.

[16] (Updated March 25, 2024). Implementation and updating of artificial intelligence clinical prediction models: a systematic review. PROSPERO identifier: CRD42024506783. https://www.crd.york.ac.uk/prospero/display_record.php?ID=CRD42024506783.

[17] Page M., McKenzie J.E., Bossuyt P.M. (2021). The PRISMA 2020 statement: an updated guideline for reporting systematic reviews. BMJ.

[18] Snell K.I.E., Levis B., Damen J.A.A. (2023). Transparent reporting of multivariable prediction models for individual prognosis or diagnosis: checklist for systematic reviews and meta-analyses (TRIPOD-SRMA). BMJ.

[19] Rethlefsen M., Kirtley S., Waffenschmidt S. (2021). PRISMA-S: an extension to the PRISMA Statement for Reporting Literature Searches in Systematic Reviews. Syst Rev.

[20] van de Schoot R., de Bruin J., Schram R. (2021). An open source machine learning framework for efficient and transparent systematic reviews. Nat Mach Intell.

[21] Collins G.S., Reitsma J.B., Altman D.G., Moons K.G. (2015). Transparent reporting of a multivariable prediction model for individual prognosis or diagnosis (TRIPOD): the TRIPOD statement. J Clin Epidemiol.

[22] Moons K.G., de Groot J.A., Bouwmeester W. (2014). Critical appraisal and data extraction for systematic reviews of prediction modelling studies: the CHARMS checklist. PLoS Med.

[23] Moons K.G.M., Wolff R.F., Riley R.D. (2019). PROBAST: a tool to assess risk of bias and applicability of prediction model studies: explanation and elaboration. Ann Intern Med.

[24] Agius R., Riis-Jensen A.C., Wimmer B. (2023). Implementing the data-driven CLL-TIM algorithm into an EPIC-based EHR: proof of concept for automated decision support models. Leuk Lymphoma.

[25] Chang C.H., Chen C.J., Ma Y.S. (2023). Real-time artificial intelligence predicts adverse outcomes in acute pancreatitis in the emergency department: comparison with clinical decision rule. Acad Emerg Med.

[26] Choi D.H., Lim M.H., Kim K.H., Shin S.D., Hong K.J., Kim S. (2023). Development of an artificial intelligence bacteremia prediction model and evaluation of its impact on physician predictions focusing on uncertainty. Sci Rep.

[27] Cronin P., Greenwald J., Crevensten G., Chueh H., Zai A. (2014). Development and implementation of a real-time 30-day readmission predictive model. AMIA Annu Symp Proc.

[28] Dontchos B., Cavallo-Hom K., Lamb L.R. (2022). Impact of a deep learning model for predicting mammographic breast density in routine clinical practice. J Am Coll Radiol.

[29] Esbenshade A.J., Zhao Z., Baird A. (2020). Prospective implementation of a risk prediction model for bloodstream infection safely reduces antibiotic usage in febrile pediatric cancer patients without severe neutropenia. J Clin Oncol.

[30] Fenn A., Davis C., Buckland D.M. (2021). Development of machine learning models to predict admission from emergency department to inpatient and intensive units. Acad Emerg Med.

[31] Giannini H.M., Ginestra J.C., Chivers C. (2019). A machine learning algorithm to predict severe sepsis and septic shock: development, implementation, and impact on clinical practice. Crit Care Med.

[32] Grout R.W., Hui S.L., Imler T.D. (2021). Development, validation, and proof-of-concept implementation of a two-year risk prediction model for undiagnosed atrial fibrillation using common electronic health data (UNAFIED). BMC Med Inf Decis Mak.

[33] de Holanda W.D., Silva L.C., Sobrinho A.A.C. (2024). Machine learning models for predicting hospitalization and mortality risks of COVID-19 patients. Expert Syst Appl.

[34] Hsu C.C., Kao Y., Hsu C.C. (2023). Using artificial intelligence to predict adverse outcomes in emergency department patients with hyperglycemic crises in real time. BMC Endocr Disord.

[35] Hulsbergen A.F.C., Lo Y.T., Awakimjan I. (2022). Survival prediction after neurosurgical resection of brain metastases: a machine learning approach. Neurosurgery.

[36] Jauk S., Kramer D., Grosauer B. (2020). Risk prediction of delirium in hospitalized patients using machine learning: an implementation and prospective evaluation study. J Am Med Inform Assoc.

[37] Jauk S., Kramer D., Stark G. (2019). Development of a machine learning model predicting an ICU admission for patients with elective surgery and its prospective validation in clinical practice. Stud Health Technol Inform.

[38] Jeon J., Yu J.Y., Song Y. (2023). Prediction tool for renal adaptation after living kidney donation using interpretable machine learning. Front Med (Lausanne).

[39] Karabacak M., Margetis K. (2023). A machine learning-based online prediction tool for predicting short-term postoperative outcomes following spinal tumor resections. Cancers (Basel).

[40] Kilpatrick C.R., Elliott M.B., Pratt E. (2014). Prevention of inpatient hypoglycemia with a real-time informatics alert. J Hosp Med.

[41] Kiss S., Pinter J., Molontay R. (2022). Early prediction of acute necrotizing pancreatitis by artificial intelligence: a prospective cohort-analysis of 2387 cases. Sci Rep.

[42] Koppes D.M., van Hees M.S.F., Koenders V.M. (2021). Nationwide implementation of a decision aid on vaginal birth after cesarean: a before and after cohort study. J Perinat Med.

[43] Kumar M., Ang L.T., Png H. (2022). Automated machine learning (AutoML)-derived preconception predictive risk model to guide early intervention for gestational diabetes mellitus. Int J Environ Res Public Health.

[44] Lazebnik T., Bahouth Z., Bunimovich-Mendrazitsky S., Halachmi S. (2022). Predicting acute kidney injury following open partial nephrectomy treatment using SAT-pruned explainable machine learning model. BMC Med Inf Decis Mak.

[45] Levin S., Barnes S., Toerper M. (2021). Machine-learning-based hospital discharge predictions can support multidisciplinary rounds and decrease hospital length-of-stay. BMJ Innov.

[46] Li Y.Y., Wang J.J., Huang S.H. (2022). Implementation of a machine learning application in preoperative risk assessment for hip repair surgery. BMC Anesthesiol.

[47] Liu M.Y., Liu C.F., Lin T.C., Ma Y.S. (2023). Implementing a novel machine learning system for nutrition education in diabetes mellitus nutritional clinic: predicting 1-year blood glucose control. Bioengineering.

[48] Liu J., Li C., Xu J., Wu H. (2018). A patient-oriented clinical decision support system for CRC risk assessment and preventative care. BMC Med Inf Decis Mak.

[49] Lupei M.I., Li D., Ingraham N.E. (2022). A 12-hospital prospective evaluation of a clinical decision support prognostic algorithm based on logistic regression as a form of machine learning to facilitate decision making for patients with suspected COVID-19. PLos One.

[50] Major V.J., Aphinyanaphongs Y. (2020). Development, implementation, and prospective validation of a model to predict 60-day end-of-life in hospitalized adults upon admission at three sites. BMC Med Inf Decis Mak.

[51] Saglietto A., Gaita F., Blomstrom-Lundqvist C. (2023). AFA-Recur: an ESC EORP AFA-LT registry machine-learning web calculator predicting atrial fibrillation recurrence after ablation. Europace.

[52] Schrempf M., Polat Erdeniz S., Kramer D. (2022). Development of an architecture to implement machine learning based risk prediction in clinical routine: a service-oriented approach. Stud Health Technol Inform.

[53] Shah N., Konchak C., Chertok D. (2020). Clinical Analytics Prediction Engine (CAPE): development, electronic health record integration and prospective validation of hospital mortality, 180-day mortality and 30-day readmission risk prediction models. PLoS One.

[54] Solomon M., Henao R., Economau-Zavlanos N. (2024). Encounter appropriateness score for you model: development and pilot implementation of a predictive model to identify visits appropriate for telehealth in rheumatology. Arthritis Care Res (Hoboken).

[55] Starr A.J., Julka M., Nethi A. (2022). Parkland trauma index of mortality: real-time predictive model for trauma patients. J Orthop Trauma.

[56] Syed A.H., Khan T. (2020). Machine learning-based application for predicting risk of type 2 diabetes mellitus (T2DM) in Saudi Arabia: a retrospective cross-sectional study. IEEE Access.

[57] Tammemägi M.C., Darling G.E., Schmidt H. (2021). Selection of individuals for lung cancer screening based on risk prediction model performance and economic factors - The Ontario experience. Lung Cancer.

[58] Wang S., Han J., Jung S.Y. (2022). Development and implementation of patient-level prediction models of end-stage renal disease for type 2 diabetes patients using fast healthcare interoperability resources. Sci Rep.

[59] Wang K., Shi Q., Sun C. (2023). A machine learning model for visualization and dynamic clinical prediction of stroke recurrence in acute ischemic stroke patients: a real-world retrospective study. Front Neurosci.

[60] Yeh C.C., Lin Y.S., Chen C.C., Liu C.F. (2023). Implementing AI models for prognostic predictions in high-risk burn patients. Diagnostics (Basel).

[61] Andaur Navarro C., Damen J., Takada T. (2021). Risk of bias in studies on prediction models developed using supervised machine learning techniques: systematic review. BMJ.

[62] Yang C., Kors J.A., Ioannou S. (2022). Trends in the conduct and reporting of clinical prediction model development and validation: a systematic review. J Am Med Inform Assoc.

[63] Andaur Navarro C., Damen J., van Smeden M. (2023). Systematic review identifies the design and methodological conduct of studies on machine learning-based prediction models. J Clin Epidemiol.

[64] Andaur Navarro C.L., Damen J.A.A., Takada T. (2022). Completeness of reporting of clinical prediction models developed using supervised machine learning: a systematic review. BMC Med Res Methodol.

[65] Collins G.S., de Groot J.A., Dutton S. (2014). External validation of multivariable prediction models: a systematic review of methodological conduct and reporting. BMC Med Res Methodol.

[66] Whittle R., Ensor J., Archer L. (Posted online June 28, 2024). Extended sample size calculations for evaluation of prediction models using a threshold for classification. Preprint.

[67] Reps J.M., Williams R.D., You S.C. (2020). Feasibility and evaluation of a large-scale external validation approach for patient-level prediction in an international data network: validation of models predicting stroke in female patients newly diagnosed with atrial fibrillation. BMC Med Res Methodol.

[68] Wong A., Otles E., Donnelly J.P. (2021). External validation of a widely implemented proprietary sepsis prediction model in hospitalized patients. JAMA Intern Med.

