# Supplementary material for: Implementation and Updating of Clinical Prediction Models: A Systematic Review
Source: Mayo Clin Proc Digit Health. 2025 May 23;3(3):100228. doi: 10.1016/j.mcpdig.2025.100228 (PMC12212251; doi:10.1016/j.mcpdig.2025.100228)
Supplement: Supplemental Appendix 3 [file mmc3.pdf]

Appendix 3: **Literature search strategy**

Database searched	Platform	Years of coverage	Records	Records after duplicates removed
Medline ALL	Ovid	1946 - Present	1026	1015
Embase	Embase.com	1971 - Present	1456	563
Web of Science Core Collection*	Web of Knowledge	1975 - Present	879	294
**Total**			**3361**	**1872**

*Science Citation Index Expanded (1975-present) ; Social Sciences Citation Index (1975-present) ; Arts & Humanities Citation Index (1975-present) ; Conference Proceedings Citation Index- Science (1990-present) ; Conference Proceedings Citation Index- Social Science & Humanities (1990-present) ; Emerging Sources Citation Index (2005-present)

No other database limits were used than those specified in the search strategies

Medline 1026

(((* Predictive Value of Tests / OR * Forecasting /) AND (Models, Statistical / OR exp * Artificial Intelligence / OR exp * Machine Learning / OR * Area Under Curve / OR exp * Algorithms / OR exp * Decision Trees /)) OR ((Predictive Value of Tests / OR Forecasting /) AND model/ AND (exp Artificial Intelligence / OR exp Machine Learning / OR Area Under Curve / OR exp Algorithms / OR exp Decision Trees /)) OR (((predict* OR forecast*) AND (model* OR machine-learn* OR deep-learning* OR neural-network* OR artificial-intelligen* OR random-forest* OR area-under-the-curve OR feature-learning OR algorithm* OR decision-tree* OR logistic-regression))).ti. OR (((predictive* OR forecast*) ADJ3 (model* OR machine-learn* OR deep-learning* OR neural-network* OR artificial-intelligen* OR random-forest* OR area-under-the-curve OR feature-learning OR algorithm* OR decision-tree* OR logistic-regression) ADJ6 (implement*)) OR ((predict* OR forecast*) ADJ3 (artificial*) ADJ3 (intelligen*)) OR ((predict* OR forecast*) ADJ3 (deep* OR machine*) ADJ3 (learning*))).ab,ti,kw.) AND (Health Plan Implementation / OR Implementation Science / OR (((implemented OR implementing OR Implementation*) ADJ3 (model*))).ab,ti,kw. OR (implement*).ti.)

Embase 1456

(((prediction/mj OR 'predictive value'/mj OR forecasting/mj) AND (model/mj OR 'artificial intelligence'/exp/mj OR 'machine learning'/exp/mj OR 'area under the curve'/mj OR algorithm/mj OR 'decision tree'/mj OR 'logistic regression analysis'/mj)) OR ((prediction/de OR 'predictive value'/de OR forecasting/de) AND model/de AND ('artificial intelligence'/exp OR 'machine learning'/exp OR 'area under the curve'/de OR algorithm/de OR 'decision tree'/de OR 'logistic regression analysis'/de)) OR 'predictive model'/de OR (((predict* OR forecast*) AND (model* OR machine-learn* OR deep-learning* OR neural-network* OR artificial-intelligen* OR random-forest* OR area-under-the-curve OR feature-learning OR algorithm* OR decision-tree* OR logistic-regression))):ti OR (((predictive* OR forecast*) NEAR/3 (model* OR machine-learn* OR deep-learning* OR neural-network* OR artificial-intelligen* OR random-forest* OR area-under-the-curve OR feature-learning OR algorithm* OR decision-tree* OR logistic-regression) NEAR/6 (implement*)) OR ((predict* OR forecast*) NEAR/3 (artificial*) NEAR/3 (intelligen*)) OR ((predict* OR forecast*) NEAR/3 (deep* OR machine*) NEAR/3 (learning*))):ab,ti,kw) AND

('implementation science'/mj OR (((implemented OR implementing OR Implementation*) NEAR/3 (model*))):Ab,ti,kw OR (implement*):ti)

Web of science 879

(TI=(((predict* OR forecast*) AND (model* OR machine-learn* OR deep-learning* OR neural-network* OR artificial-intelligen* OR random-forest* OR area-under-the-curve OR feature-learning OR algorithm* OR decision-tree* OR logistic-regression))) OR TS=(((predictive* OR forecast*) NEAR/2 (model* OR machine-learn* OR deep-learning* OR neural-network* OR artificial-intelligen* OR random-forest* OR area-under-the-curve OR feature-learning OR algorithm* OR decision-tree* OR logistic-regression) NEAR/5 (implement*)) OR ((predict* OR forecast*) NEAR/2 (artificial*) NEAR/2 (intelligen*)) OR ((predict* OR forecast*) NEAR/2 (deep* OR machine*) NEAR/2 (learning*)))) AND (TS=(((implemented OR implementing OR Implementation*) NEAR/2 (model*))) OR TI=(implement*)) AND TS=((health-care OR healthcare OR hospital* OR medical* OR mortalit* OR surviv* OR disease* OR surger* OR therap* OR treatment* OR drug*))
