# Supplementary material for: Implementation and Updating of Clinical Prediction Models: A Systematic Review
Source: Mayo Clin Proc Digit Health. 2025 May 23;3(3):100228. doi: 10.1016/j.mcpdig.2025.100228 (PMC12212251; doi:10.1016/j.mcpdig.2025.100228)
Supplement: Supplemental Appendix 4 [file mmc4.pdf]

Appendix 4: **List of items extracted**

	TRIPOD	PROBAST	CHARMS	Other reason
**DOI**				For referencing purposes
**First Author**				For referencing purposes
**Journal**				For referencing purposes
**Impact factor**				To look into whether these implementations are published in high impact journals
**Type of article**				Identifying the study design
**Publication year**			X	
**Country, model development and validation**			X	
**Country, external validation**			X	
**Country, implementation**			X	
**Clinical field**				To look into whether models are implemented more in certain clinical fields
**Study setting**			X	
**Number of centers**			X	
**Objective**				PRISMA requirement
**Intended user**				Some user groups might be underrepresented
**Use of multidisciplinary team**				Might be an important factor in getting models implemented
**Data source, model development and internal validation**		X	X	
**Mean age, model development and internal validation**	X			
**Female, model development and internal validation**	X			
**Mean BMI, model development and internal validation**	X			

**External validation (yes/no)**	**X**			
**Data source, external validation**		**X**	**X**	
**Mean age, external validation**	**X**			
**Female, external validation**	**X**			
**Mean BMI, external validation**	**X**			
**Impact assessment design**				Look into what designs are used for impact assessments
**Improvement of patient care (Yes/No)**				Assess whether analysis showed improvement in patient care
**Description of how patient care was improved**				Assess whether analysis showed improvement in patient care
**Study period, model development and internal validation**			**X**	
**Study period, external validation**			**X**	
**Study period, impact assessment**			**X**	
**Target population (eligibility criteria)**			**X**	
**Outcome**			**X**	
**Modeling method**			**X**	
**Sample size (N), development and internal validation**			**X**	
**Sample size (N), external validation**			**X**	
**Sample size (N), impact assessment**			**X**	

**Observation window (days)**				Design of the model
**Time-at-risk period (days)**				Design of the model
**Outcome events (%), model development and internal validation**		**X**		
**Number of candidate predictors**			**X**	
**Predictor selection**			**X**	
**Number of predictors**	**X**	**X**	**X**	
**Number of continuous predictors**			**X**	
**Continuous predictors (%)**			**X**	
**Reporting of missing data percentage (yes/no)**			**X**	
**Complete cases (%)**			**X**	
**Handling of missing data**	**X**	**X**	**X**	
**Imputation (yes/no)**	**X**	**X**	**X**	
**Imputation method**	**X**	**X**	**X**	
**Code availability (yes/no)**	**X**			
**Discrimination, internal validation (95% CI)**	**X**	**X**	**X**	
**Discrimination, external validation (95% CI)**	**X**	**X**	**X**	
**Calibration, internal validation (yes/no)**	**X**	**X**	**X**	
**Means of calibration, internal**	**X**	**X**	**x**	

**validation (e.g. calibration plot)**				
**Calibration, external validation (yes/no)**	X	X	X	
**Means of calibration, external validation (e.g. calibration plot)**	X	X	X	
**Other performance metric, internal validation (95% CI)**				e.g. AUPRC and decision curve analysis
**Other performance metric, external validation (95% CI)**				e.g. AUPRC and decision curve analysis
**Discrimination, implementation (95% CI)**			X	Also looked into this for internal and external validation
**Calibration, implementation**			X	Also looked into this for internal and external validation
**Other performance metric, implementation (95% CI)**				e.g. AUPRC and decision curve analysis
**Means of implementation**				Features relevant to evaluation of means of implementation
**Covariate conversion (yes/no)**				Features relevant to evaluation of updating methods
**Local updating before implementation (yes/no)**				Features relevant to evaluation of updating methods
**Updating (yes/no)**				Features relevant to evaluation of updating methods
**Updating method(s)**				Features relevant to evaluation of updating methods
**Time to updating**				Features relevant to evaluation of updating methods
**RoB judgement PROBAST Domain 1: Participants**		X		

Were appropriate data sources used, e.g. cohort, RCT or nested case-control study data?	X
Were all inclusions and exclusions of participants appropriate?	X
RoB judgement PROBAST Domain 2: Predictors	X
Were predictors defined and assessed in a similar way for all participants?	X
Were predictor assessments made without knowledge of outcome data?	X
Are all predictors available at the time the model is intended to be used?	X
RoB judgement PROBAST Domain 3: Outcome	X
Was the outcome determined appropriately?	X
Was a pre-specified or standard outcome definition used?	X
Were predictors excluded from the outcome definition?	X
Was the outcome	X

defined and determined in a similar way for all participants?		
Was the outcome determined without knowledge of predictor information?	X	
Was the time interval between predictor assessment and outcome determination appropriate?	X	
RoB judgement PROBAST Domain 4: Analysis	X	
Was there a reasonable number of participants with the outcome?	X	
Were continuous and categorical predictors handled appropriately?	X	
Were all enrolled participants included in the analysis?	X	
Were participants with missing data handled appropriately?	X	
Was selection of predictors based on univariable analysis avoided?	X	
Were complexities in	X	

**the data (e.g., censoring, competing risks, sampling of control participants) accounted for appropriately?**	
**Were relevant model performance measures evaluated appropriately?**	**X**
**Were model overfitting and optimism in model performance accounted for?**	**X**
**Do predictors and their assigned weights in the final model correspond to the results from the reported multivariable analysis?**	**X**
**Overall judgement about RoB**	**X**

BMI=Body Mass Index.
