# Supplementary material for: Implementation and Updating of Clinical Prediction Models: A Systematic Review
Source: Mayo Clin Proc Digit Health. 2025 May 23;3(3):100228. doi: 10.1016/j.mcpdig.2025.100228 (PMC12212251; doi:10.1016/j.mcpdig.2025.100228)
Supplement: Supplemental Appendix 5 [file mmc5.pdf]

PRISMA 2020 flow diagram for new systematic reviews which included searches of databases and registers only

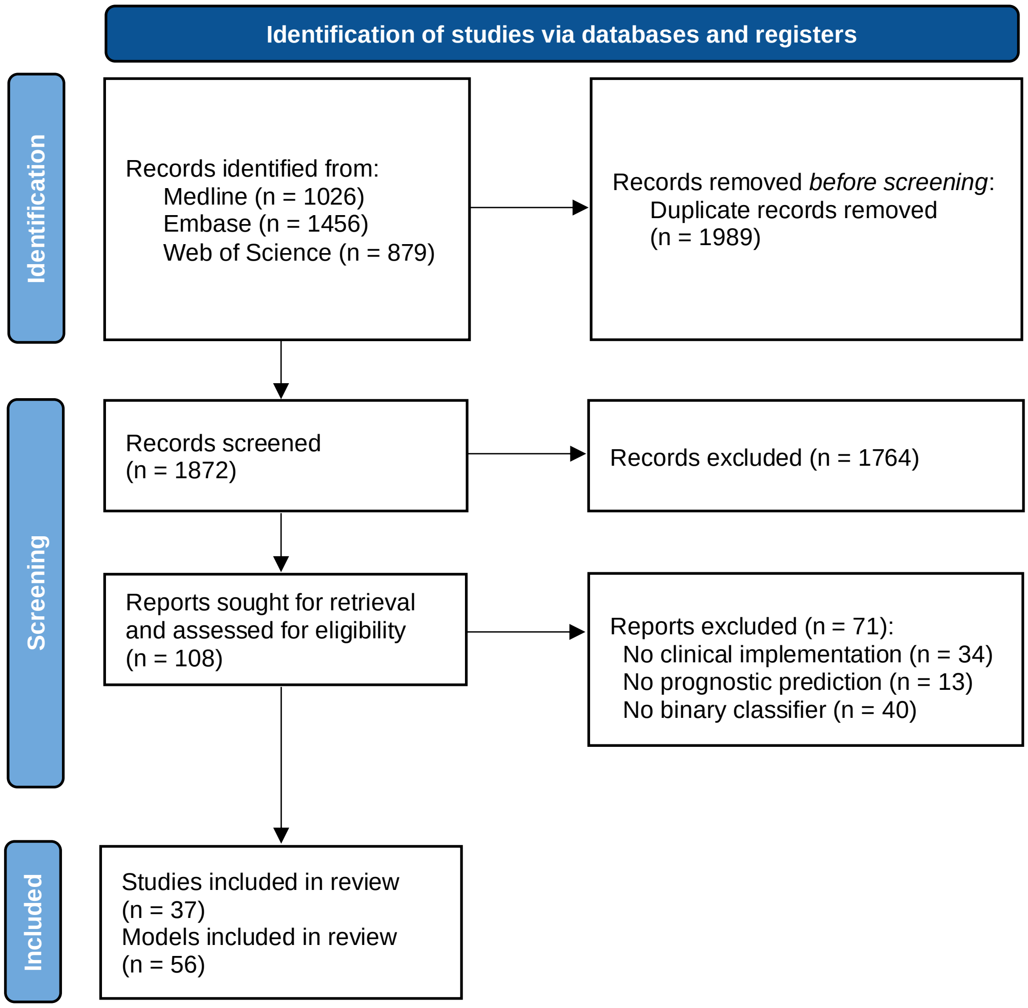

*Some reports had multiple reasons for exclusion. Therefore the number of articles per reason for exclusion will exceed the total number of excluded reports.
