# Supplementary material for: Implementation and Updating of Clinical Prediction Models: A Systematic Review
Source: Mayo Clin Proc Digit Health. 2025 May 23;3(3):100228. doi: 10.1016/j.mcpdig.2025.100228 (PMC12212251; doi:10.1016/j.mcpdig.2025.100228)
Supplement: Supplemental Appendix 6 [file mmc6.pdf]

***Appendix 6:* List of excluded studies at full-text screening stage, with brief reasons**

Study	Clinically implemented	Prognostic prediction	Binary classifier
Amutha et al (2018)	Yes	No	No
Astley et al (2024)	No	Yes	No
Bauer et al (2020)	No	Yes	Yes
Belmin et al (2022)	Yes	Yes	No
Bhandarkar et al (2023)	Yes	No	Yes
Biehl et al (2023)	Yes	Yes	No
Callender et al (2023)	No	Yes	Yes
Chae et al (2021)	Yes	Yes	No
Cheng et al (2023)	Yes	Yes	No
Connell et al (2021)	Yes	Yes	No
Cortigiani et al (2022)	Yes	Yes	No
Cui et al (2022)	No	Yes	Yes
Cupido et al (2023)	No	Yes	Yes
Dadabhoy et al (2023)	No	Yes	Yes
Dahella et al (2020)	Yes	Yes	No
Davis et al (2022)	No	Yes	Yes
Dey et al (2018)	No	Yes	Yes
Dhondt et al (2022)	No	No	No
Dominguez et al (2021)	Yes	No	Yes
Dugard et al (2017)	No	Yes	Yes
Ehrig et al (2023)	Yes	No	Yes
Groezinger et al (2020)	Yes	No	Yes
Grootes et al (2024)	Yes	Yes	No
Hamm et al (2021)	No	Yes	Yes
Han et al (2020)	No	Yes	No
Hoban et al (2017)	No	Yes	Yes
Huang et al (2022)	No	Yes	Yes
Ismaeel et al (2015)	Yes	Yes	No
Kanda et al (2023)	Yes	Yes	No
King et al (2022)	Yes	Yes	No
Leonard et al (2022)	No	Yes	Yes
Liu et al (2021)	Yes	Yes	No
Lodise et al (2019)	Yes	Yes	No
Lustberg et al (2016)	No	Yes	No
Marcovitz et al (2016)	No	No	Yes
Martinez et al (2017)	No	No	No
Massoud et al (2023)	No	Yes	Yes
Mittendorf et al (2022)	No	Yes	Yes
Mohammed et al (2022)	No	Yes	Yes
Murtas et al (2021)	Yes	Yes	No
Nasrat et al (2022)	No	Yes	Yes
Nayshool et al (2022)	Yes	Yes	No
Nguyen et al (2021)	No	Yes	Yes
Nilius et al (2023)	No	No	Yes
Ninomiya et al (2023)	No	Yes	Yes
Ofoma et al (2014)	Yes	Yes	No
Oliver at al (2021)	Yes	Yes	No
Padrik et al (2023)	No	Yes	Yes
Park et al (2021)	No	Yes	No
Rabbani et al (2023)	Yes	Yes	No

Rathina et al (2023)	No	Yes	Yes
Rathod et al (2021)	No	Yes	Yes
Rogasch et al (2023)	Yes	Yes	No
Ryu et al (2023)	No	No	No
Sakman et al (2022)	Yes	Yes	No
Sankaranarayanan et al (2021)	No	Yes	No
Secher et al (2022)	Yes	No	No
Seevaratnam et al (2024)	Yes	Yes	No
Sendak et al (2020)	Yes	Yes	No
Shahid et al (2022)	Yes	Yes	No
Shanbehzadeh et al (2022)	No	Yes	Yes
Studerus et al (2020)	Yes	Yes	No
Syed et al (2022)	Yes	No	Yes
Tan et al (2017)	Yes	Yes	No
Van den Berg et al (2023)	No	Yes	No
Van Dijk et al (2023)	Yes	Yes	No
Van Es et al (2023)	Yes	No	Yes
Vara Prasada Rao et al (2022)	No	Yes	Yes
Wilson et al (2023)	Yes	Yes	No
Wong et al (2021)	Yes	Yes	No
Xia et al (2023)	Yes	Yes	No
