# Supplementary material for: Implementation and Updating of Clinical Prediction Models: A Systematic Review
Source: Mayo Clin Proc Digit Health. 2025 May 23;3(3):100228. doi: 10.1016/j.mcpdig.2025.100228 (PMC12212251; doi:10.1016/j.mcpdig.2025.100228)
Supplement: Supplemental Appendix 7 [file mmc7.pdf]

***Appendix 7:* Demographics of the data used to develop and validate the included models (n=57)**

	**Mentioned***		**Not mentioned***
	Mean (SD)	Median (IQR)	Missing (N, %)
**Demographics**			
Model development and internal validation			
Sample size		4,448 (25,122)	
Age	56·3 (10·3)		29 (52)
%Female	51·1 (20·0)		13 (23)
BMI	24·9 (1·9)		40 (71)
External validation			
Sample size		7,341 (79,683)	1 (7)
Age	60·5 (7·2)		13 (87)
Female	51·4 (31·7)		8 (53)
BMI	28		14 (93)

SD=Standard deviation, IQR=Interquartile range, Body Mass Index (BMI) in kg/m^2

*Mentioned in either the model development and internal validation, external validation or implementation article of the included models
