# Supplementary material for: Implementation and Updating of Clinical Prediction Models: A Systematic Review
Source: Mayo Clin Proc Digit Health. 2025 May 23;3(3):100228. doi: 10.1016/j.mcpdig.2025.100228 (PMC12212251; doi:10.1016/j.mcpdig.2025.100228)
Supplement: Supplemental Appendix 8 [file mmc8.pdf]

***Appendix 8:* Tabular presentation for PROBAST results**

	RoB Domains				Overall RoB
	Participants	Predictors	Outcome	Analysis	
Agius[24]	+	+	+	-	-
Chang[25]	-	+	+	-	-
Choi[26]	-	+	+	?	?
Cronin[27]	-	+	+	-	-
Dontchos[28]	+	+	+	?	?
Ebenshade[29]	-	+	+	-	-
Fenn[30]	-	+	+	?	-
Giannini[31]	-	+	+	-	-
Grout[32]	+	+	+	-	-
Holanda[33]	-	+	+	-	-
Hsu[34]	-	+	+	+	-
Hulsbergen[35]	-	+	+	+	-
Jauk ('19)[36]	-	+	+	-	-
Jauk ('20)[37]	-	+	+	-	-
Jeon[38]	-	+	+	-	-
Karabacak[39]	+	+	+	-	-
Kilpatrick[40]	-	+	+	-	-
Kiss[41]	+	+	+	-	-
Koppes[42]	+	+	+	+	+
Kumar[43]	+	+	+	-	-
Lazebnik[44]	-	+	+	-	-
Levin[45]	-	+	+	-	-
Li[46]	-	+	+	-	-
Liu ('18)[48]	-	+	+	-	-
Liu ('23)[47]	-	+	+	-	-
Lupei[49]	?	+	+	-	-
Major[50]	-	+	+	-	-
Saglietto[51]	+	+	+	+	+
Schrempf[52]	-	+	+	+	-
Shah[53]	-	+	+	-	-
Solomon[54]	-	+	+	-	-
Starr[55]	-	+	+	-	-
Syed[56]	-	+	+	-	-
Tammemägi[57]	-	+	+	?	-
Wang ('22)[58]	-	+	+	?	-
Wang ('23)[59]	-	+	+	-	-
Yeh[60]	-	+	+	-	-

PROBAST=Prediction model Risk of Bias Assessment Tool. RoB=Risk of Bias.
Applicability is omitted as this systematic review is performed across all medical domains.
+ indicates a low risk of bias. - indicates a high risk of bias. ? indicates an unclear risk of bias.
