# Supplementary material for: Implementation and Updating of Clinical Prediction Models: A Systematic Review
Source: Mayo Clin Proc Digit Health. 2025 May 23;3(3):100228. doi: 10.1016/j.mcpdig.2025.100228 (PMC12212251; doi:10.1016/j.mcpdig.2025.100228)
Supplement: Supplemental Appendix 9 [file mmc9.pdf]

***Appendix 9:* Included impact assessments**

Impact assessment	Improvement in patient care (Yes/No)	Description of main results
Choi et al[26]	Yes	Significant improvement in AUC (0·639 to 0·703), especially in cases of high physician uncertainty and low model uncertainty
Dontchos et al [28]	Yes	Reduced radiologist variability of dense mammogram classification
Giannini et al[31]	Yes	Reduction in median time-to ICU transfer (16 to 8 hours)
Kilpatrick et al[40]	Yes	Significant decrease by 68% in the rate of severe hypoglycemia in alerted high-risk patients versus non-alerted high-risk patients
Koppes et al [42]	Yes	Significant reduction in practice variation (SD 0·07) Clinically relevant reduction (21%) in overall complication rate.
Levin et al[45]	Yes	Reduction in hospital length-of-stay of over 12 hours on an internal medicine and telemetry unit.
Li et al[46]	Yes	Superior AUC scores compared to standard care (ASA-PS), when it comes to composite adverse outcomes (0·776 vs 0·629), ICU admission (0·844 vs 0·629) and PLOS (0·854 vs 0·618)
Agius et al[24]	No	The implemented version of CLL-TIM achieved similar performance within the real-time production environment when compared to the original research environment
Chang et al[25]	No	The group with the use of AI prediction had lower rates of sepsis (1.67% vs. 2.62%) and mortality (0.83% vs 2.62%). However, there was no statistical significance in both sepsis (p = 0.76) and mortality (p = 0.349).
Cronin et al[27]	No	The model developed retrospectively had an AUC of 0.705 with good calibration. The real-time implementation had an AUC of 0.671 although the model was overestimating readmission risk.
Ebenshade et al[29]	No	On detecting BSI status, the EsVan, EsVan2, and EsVan2b models achieved an AUC/C-index of 0.802,0.824, and 0.818, respectively. Consistent with previous validations results, the models performed even better in detecting high-risk BSIs, achieving an AUC/C-index of 0.837, 0.855, and 0.858, respectively.
Fenn et al[30]	No	The models had similar performance in both the academic- and community-based settings as well as across the 2019 and real-time encounter data.
Grout et al[32]	No	In the EHR pilot, 7916/22,272 (35.5%; mean age, 66 years; female 50%) were identified as higher risk for AF; 5582 (70%) had CHA2DS2-VASc score ≥ 2.
Hsu et al[34]	No	No significant differences in ICU

		admission or all-cause mortality were detected between the non-AI and AI groups.
Jauk et al (2020)[36]	No	Discrimination on prospective data (AUC 0.86) was as good as in the test dataset, but calibration was poor. The predictions correlated strongly with delirium risk perceived by experts in the blinded (r=0.81) and nonblinded (r=0.62) settings.
Jauk et al (2019) [37]	No	In the prospective implementation, the model achieved a sensitivity of 73.3% and a specificity of 80.8%.
Lupei et al[49]	No	Validation in the PUI population had an AUC of 0.82 (95%-CI: 0.81, 0.83). The ED CDS system performed well in real-time with an AUC of 0.85 (95%-CI, 0.83, 0.87). Zero patients in the lowest quintile developed “severe” COVID-19. Patients in the highest quintile developed “severe” COVID-19 in 33.2% of cases.
Major et al[50]	No	Applied prospectively for nine months, 41,728 predictions were generated in real-time (median [IQR], 1.3 [0.9, 32] minutes). An operating criterion of 75% positive predictive value identified 104 predictions at very high risk (0.25%) where 65% (50 from 77 well-timed predictions) led to death within 60 days.
Shah et al[53]	No	Models were derived and validated at three time points: retrospective, prospective at discharge, and prospective at 4 hours after presentation. AUCs of hospital mortality were 0.91,0.89 and 0.77, respectively. AUCs for 30-day readmission were 0.71, 0.71 and 0.69.
Solomon et al[54]	No	Providers were more likely to agree with true versus artificial recommendations (Cohen’s κ = 0.45, P < 0.001), and the model increased the number of appropriate telehealth visits.
Starr et al[55]	No	During the first year of clinical use, when used in 776 patients, the last PTIM score accurately predicted 20 of the twenty-three 12-hour time intervals within 48 hours of mortality, for sensitivity of 86.9% (95% CI, 73%–100%). The specificity was 94.7% (95% CI, 93%–96%), and the positive predictive value was 33.3% (95% CI, 21.4%–45%). The model predicted survival for 716 time intervals and was incorrect 3 times, yielding a negative predictive value of 99.6% (95% CI, 99.1%–100%). The area under the curve of the receiver operating characteristic curve was 0.97.
Tammemägi et al[57]	No	The Pilot cancer detection rate was significantly higher than in the NLST (p = 0.009) or NELSON (p = 0.003) and there was a significant shift to early stage compared to historical Ontario Cancer Registry statistics (p < 0.0001). Pre- and post-Pilot evaluations found that conducting quality risk assessments were not excessively time consuming or difficult, and participants’ satisfaction was high.
Yeh et al[60]	No	AI models have higher values for all indicators than Baux score models. AI models of prolonged hospital stay and

	overall adverse effects outperformed Baux score models in a statistically significant manner ($p < 0.05$).

ASA-PS=American Society of Anesthesiologist-Physical Status, AUC=Area Under the Curve, BSI=Bloodstream infection, CLL-TIM=Chronic Lymphocytic Leukemia Treatment Infection Model, ED CDS = emergency department clinical decision support. PLOS=Prolonged length of hospital stay, PTIM = Parkland Trauma Index of Mortality. PUI = person under investigation ICU=Intensive Care Unit, PLOS=Prolonged length of hospital stay.
